# 

**Published:** 1883-09

**Authors:** 


					﻿Vol. HI.
SEPTEMBER, 1883,
No. 9.
THE
HOMEOPATHIC pH^lClAH,
A MONTHLY JOURNAL OF MEDICAL SCIENCE.
“ Magna est veritas, el prtevalebit.”
_t±J. CT. T i H {' H jj ZLZE. I J -j
o	EDITOR.
CONTENTS:
(See inside of Cover.)
PHILADELPHIA:
No. 3109 CHESTNUT STREET.
□STEl-W YORK:
A. L. CHATTERTON, PUBLISHING^ COMPANY, Publisher’s Agents.
X. O 3ST JD O TT :
ALFRED HEATH & CO., Sole Agents for Great Britain,
114 Ebury Street, Eaton Square.
Yearly Subscription, $2.50, invariably in advance.
Entered at the Philadelphia Post-Office as Second- Class Mail Matter.
				

